# Masturbation in the Young Girl the Cause of Acquired Sexual Perversion

**Published:** 1905-12

**Authors:** William Lee Howard

**Affiliations:** Baltimore, M. D.


### Masturbation in the Young Girl the Cause of Acquired Sexual Perversion.

By WILLIAM LEE HOWARD, M. D., Baltimore, M. D.

TO the specialist in functional neuroses is revealed the true personality, the real ego, of individuals. Few surgeons or general practitioners realize what a great role autoerotic phenomena play in the development of the young girl, or the influence it has on her future. I have no desire to be considered as giving expression to the idea that every young girl has autoerotic periods, or that in many girls excessive eroticism is the cause of those puzzling conditions met with in general practice and put down in the casebook and treated as hysteria. What loopholes that word hysteria offers the physician!

The idea I wish to express is, that the psychic side of life, and the basis of life,—sexual activity,—is not sufficiently understood or studied by the physician. Many seem afraid to admit the passional element of humanity, or else are blind to its tremendous ethical significance.

The man who attempts to get at the cause of his patient's anaemia by blood count and examination, by all the objective signs his laboratory and clinical experience has taught him, generally discovers what he is after, but not always. It does not occur to him that the young patient has at this period a very active psychic side of life as well as a physiologic one. The girl may be, and generally is, innocent of the cause of her trouble, but not ignorant of the teasing, wakeful auto-erotic phenomena which have disturbed her normal physiologic rythms.

In normal children there are great variations in the age of experiencing voluptuous sensations before puberty. Masturbation has not up to this period been practiced, but unless the case is understood—and this comprehension must be accomplished without giving any suggestive ideas or questions—the habit will soon be formed. It is these cases that gradually worm into nymphomania or some variant of sexual activity.

The following case is an example of the insidious and baneful effect of early masturbation following the autoerotic day dreams of a young girl.

Mrs. M., age 44. She was sent to me by Dr. Morrison. It took several days to get a satisfactory history of the woman as she had heretofore only been obliged to answer the few stereotyped questions put to her by various physicians.

She married when she was twenty-four years of age a business man five years her senior. They lived together for five years, when her husband left her and went abroad to live. The reason he left was on account of his wife preferring self-manustupration to normal intercourse. When her husband did have sexual relations with her she experienced no psychic or physiologic orgasm, but would compel him after the act to leave the room while she satisfied herself by masturbation.

Her present condition is as follows: Physically she is large, fat and flabby. She is neurotic in her conversation and actions. She lives alone, although financially able to keep a household of servants. Menstruation is regular, and there are no subjective signs of approaching menopause. The impulse to masturbate has undergone considerable change from its earlier origin. This woman will pass a man on the street in the evening, —she seldom goes out during the day,—and touching him with her hand or elbow, will immediately have an uncontrolable impulse to masturbate, which impulse she will immediately gratify by rushing up an alley, or into the closet of the first house, or even a saloon, to which she can gain access. If she has this impulse in the theatre she rushes to the toilet room. So uncontrolable has this morbid desire become that she often masturbated under the sheltering shades of a street corner, or any place offering the slightest idea of temporary seclusion.

This woman was born in New England of the class of puritanical parents, whose sole ideas of bringing up their children appears to be one of ignoring everything connected with the physiology and hygiene of life. Such children are left to the instruction of other wiser but less innocent children and soon become the innocent victims of sexual neuropaths and perverts. Having had no instruction or advice in sexual matters these children easily become the placid scholars and imitators of the sly and insinuating erotopath.

As I have in my work on this subject gone deeply into this vitally important matter, it is unnecessary here to comment further.

At fourteen years of age our subject was sent to a boarding school some distance from her Vermont home. On the journey to the school the train became snowbound, and as night approached the passengers were informed that they would have to remain over in the small town where the train was stalled. The young girl's embarassment and fear was noticed by a woman passenger, who apparently took a motherly interest in the child.

The rest of the story is soon told. That night, the weak, undeveloped sexual cells of the cortex were awakened—directed in the wrong channel, and a child masturbator with psychic imaginings and fancies of women constantly arising, was the

1. The Perverts, Dillingham Co., N. Y.

result. These inverted pictures kept up until the woman reached the age of about thirty, when the condition now present gradually made its appearance.

In this case we have an undoubted case of inversion through acquirment. There are none of the signs, objective or subjective, of the congenital invert. Properly taught in early childhood of the significance of sexual activity and its normal relation to health, this unfortunate girl would have become a happy wife and devoted mother.

Where was the family physician when it was decided to send this little girl among strangers? A full knowledge of the moral sin he committed when he ignored the most important advice he should have given mother and father, would be a just punishment for him. No man whose moral standards are primarily conventional can understand the power of certain impulses; nor can the man whose perceptions of moral values is uncertain or blurred, estimate his fearful responsibility in treating children of psychopathic or eropathic tendencies. A clear moral, as well as a worldly and physiologic insight, are the necessary requisites for the physician who expects success among those exhibiting excessive normal or morbid impulses.

1126 North Calvert Street.

